# miR-16 targets fibroblast growth factor 2 to inhibit NPC cell proliferation and invasion via PI3K/AKT and MAPK signaling pathways

**DOI:** 10.18632/oncotarget.6504

**Published:** 2015-12-08

**Authors:** Qingmei He, Xianyue Ren, Jiewei Chen, Yingqin Li, Xinran Tang, Xin Wen, Xiaojing Yang, Jian Zhang, Yaqin Wang, Jun Ma, Na Liu

**Affiliations:** ^1^ Sun Yat-Sen University Cancer Center, State Key laboratory of Oncology in South China, Collaborative Innovation Center of Cancer Medicine, Guangzhou, People's Republic of China

**Keywords:** miR-16, fibroblast growth factor 2, nasopharyngeal carcinoma, tumor growth, metastasis

## Abstract

Dysregulation of miRNAs has been shown to contribute to the carcinogenesis and progression of nasopharyngeal carcinoma (NPC). Our previous microarray data showed that miR-16 expression is significantly decreased in archived NPC tissues. Here, we confirmed that miR-16 was reduced in NPC cell lines and freshly-frozen samples. Ectopic expression of miR-16 suppressed NPC cell proliferation, migration, and invasion *in vitro* and inhibited tumor growth and metastatic colonization in the lung *in vivo*. Furthermore, *fibroblast* growth factor 2 (*FGF2*) was identified as a direct target of miR-16, and both *phosphoinositide-3- kinase/AKT* (*PI3K/AKT*) and *mitogen-activated protein kinase* (*MAPK*) signaling pathways were repressed after miR-16 overexpression. In addition, the restoration of *FGF2* reversed the suppressive effects of miR-16. Together, these results indicated that miR-16 suppresses NPC carcinogenesis and progression by targeting *FGF2*, thereby representing a potential target for miRNA-based therapy for NPC in the future.

## INTRODUCTION

MicroRNAs (miRNAs) are a family of highly conserved short non-coding RNAs that can regulate gene expression by base pairing with the 3′-untranslated region (3′-UTR) to enhance mRNA degradation or inhibit post-transcriptional translation [1]. Emerging evidence indicates that miRNAs are abnormally expressed in a variety of human cancers, and the dysregulation of miRNAs contributes to tumor initiation, promotion, and progression [2–4].

Nasopharyngeal carcinoma (NPC) is a malignant tumor with the highest incidence rate in Southern China, especially in Guangdong and Hong Kong [5]. Local recurrence and distant metastasis are two major reasons for treatment failure and NPC-related death [6]. Abnormal expression of miRNAs in NPC has been reported [7–9], and several dysregulated miRNAs, such as miR-29c, miR-34c, miR-93, miR-143, miR-451, miR-504, and miR-744, can regulate NPC cell growth, proliferation, invasion, and metastasis [10–16]. These findings indicate that the dysfunction of miRNAs may be involved in NPC carcinogenesis and progression. Clearly, further investigation is required to clarify the roles of miRNAs in NPC tumorigenesis and to identify miRNAs that may serve as novel treatment targets for NPC patients.

Our previous microarray data indicated that miR-16 expression is significantly decreased in archived NPC tissues [7]. Recent studies have demonstrated that miR-16 acts as tumor suppressor by affecting cell apoptosis, the cell cycle, cell proliferation, and invasion in various types of cancers, such as chronic lymphocytic leukemia, prostate cancer, ovarian cancer, lung cancer, and gastric cancer [17–26]. Investigations have also suggested that miR-16 could serve as prognostic biomarker and enhance chemosensitivity, and the systemic delivery of miR-16 represents a novel treatment approach [27–32]. *Fibroblast growth factor (FGF)/fibroblast growth factor receptor (FGFR)* signaling axis plays important roles in driving tumor progression [33]. *FGF2*, which often localizes to the nucleus and/or cytoplasm, belongs to the *FGF* family and regulates the tumorigenesis and progression of a variety of cancers [34–35].

Here, we investigated the role and mechanism of miR-16 in NPC tumorigenesis and progression. We confirmed that miR-16 was decreased in NPC and that ectopic expression of miR-16 suppressed NPC cell proliferation, migration, and invasion *in vitro* and *in vivo*. *FGF2* was verified as a direct functional target of miR-16, and both the *PI3K/AKT* and *MAPK* signaling pathways were repressed by restoration of miR-16. Taken together, these results suggested that miR-16 suppresses NPC progression by targeting *FGF2*, thus representing a potential target for miRNA-based therapy for NPC.

## RESULTS

### miR-16 is decreased in NPC tissues and cell lines

**Figure 1:**
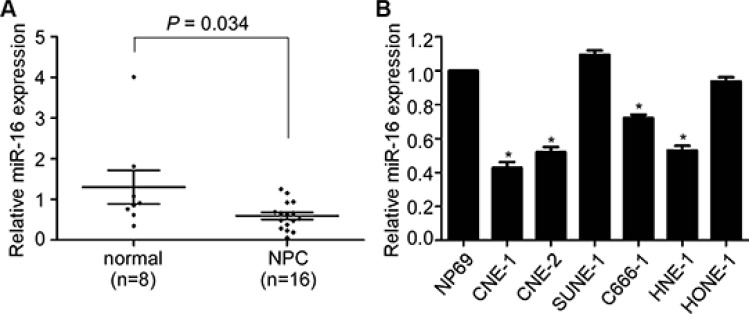
miR-16 is decreased in NPC tissues and cell lines (**A**) Relative miR-16 expression in NPC tissues (*n =* 16) and normal nasopharyngeal epithelial tissues (*n =* 8). (**B**) Relative miR-16 expression in six NPC cell lines and the immortalized normal nasopharyngeal epithelial cell, NP69. U6 was used as an endogenous control. The data are presented as the mean ± S.D.; *p* values were calculated using Student's *t*-test.

Based on our previous microarray data, miR-16 was found to be significantly decreased in archived NPC tissues. To validate this result, we first detected the miR-16 expression level in 16 freshly frozen NPC and 8 normal nasopharyngeal epithelial tissue samples using quantitative RT-PCR. The overall average expression level of miR-16 in NPC specimens was decreased by 46% compared with the normal nasopharyngeal epithelial tissue samples (fold change = 0.46, *p* = 0.034, Figure 1A). We then examined the expression level of miR-16 in six NPC cell lines and the immortalized nasopharyngeal epithelial cell line, NP69. Similarly, the miR-16 expression was significantly decreased (0.43 to 0.72 fold) in most NPC cell lines (except for SUNE-1 and HONE-1) compared with the NP69 cell line (Figure 1B). These results suggested that miR-16 is downregulated in NPC and that it may be involved in NPC tumorigenesis and progression.

### miR-16 suppresses NPC cell viability, cell proliferation, and tumor growth

**Figure 2:**
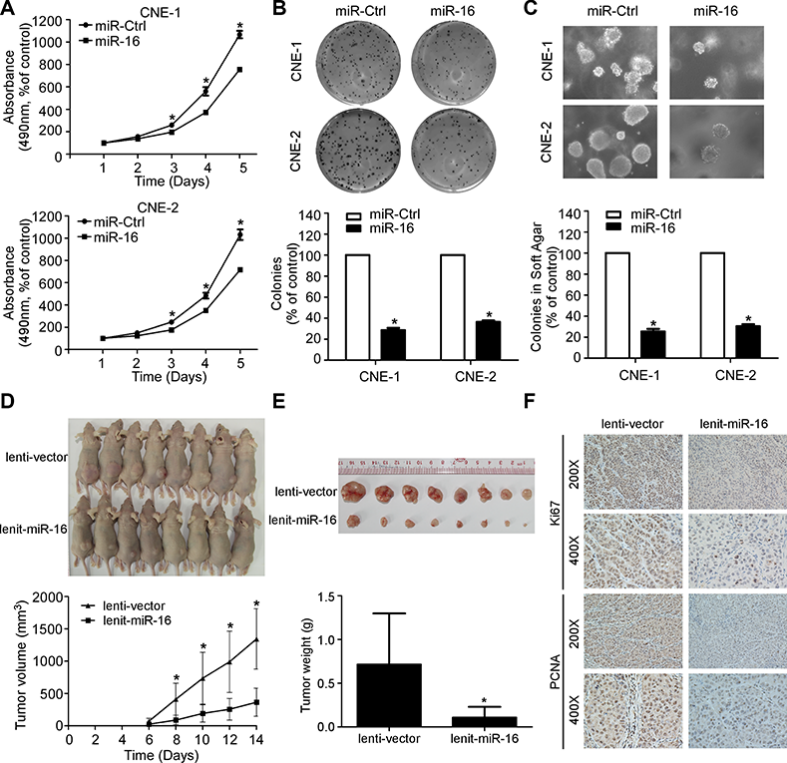
miR-16 suppresses NPC cell viability, cell proliferation, and tumor growth (**A**) Cell viability of the indicated NPC cells was examined using MTT assays. (**B–C**) Representative images and quantification of colonies of the indicated cells tested by colony formation (B) and anchorage-independent soft-agar assays (C). (**D–F**) Xenograft tumor growth models in nude mice were constructed, and representative images of the formed tumors and the growth curves of tumor volumes (D) as well as of the excised tumors and tumor weights (E) are shown. Immunohistochemistry staining showed that miR-16 overexpression suppressed NPC cell proliferation *in vivo* as indicated by the expression of Ki67 and PCNA (F). The data are presented as the mean ± S.D.; *p* values were calculated using Student's *t*-test.

To evaluate whether the ectopic expression of miR-16 affects the viability and proliferation of NPC cells, we transiently transfected CNE-1 and CNE-2 cells with miR-16 mimics and performed MTT, colony formation, and anchorage-independent soft-agar assays. Ectopic expression of miR-16 in NPC cells remarkably inhibited the cell growth and colony formation rates, as determined by MTT and colony formation assays, respectively, demonstrating the suppressive effect of miR-16 on cell proliferation, which was further confirmed by anchorage-independent soft-agar assays (Figure 2A–2C). Furthermore, we established CNE-1 cell lines stably expressing miR-16 (lenti-miR-16) or control empty vector (lenti-vector) and examined the effect of miR-16 on tumor growth using a xenograft tumor growth model. As shown in Figure 2D and 2E, the tumors formed by lenti-miR-16 cells had smaller volumes and lower weights than those formed by lenti-vector cells. Meanwhile, the lenti-miR-16 tumors showed lower percentage of Ki67- and PCNA-positive cells compared with the lenti-vector tumors (Figure 2F). These data demonstrated that miR-16 can inhibit NPC cell proliferation and tumor growth.

### miR-16 inhibits NPC cell migration, cell invasion, and lung metastatic colonization

**Figure 3:**
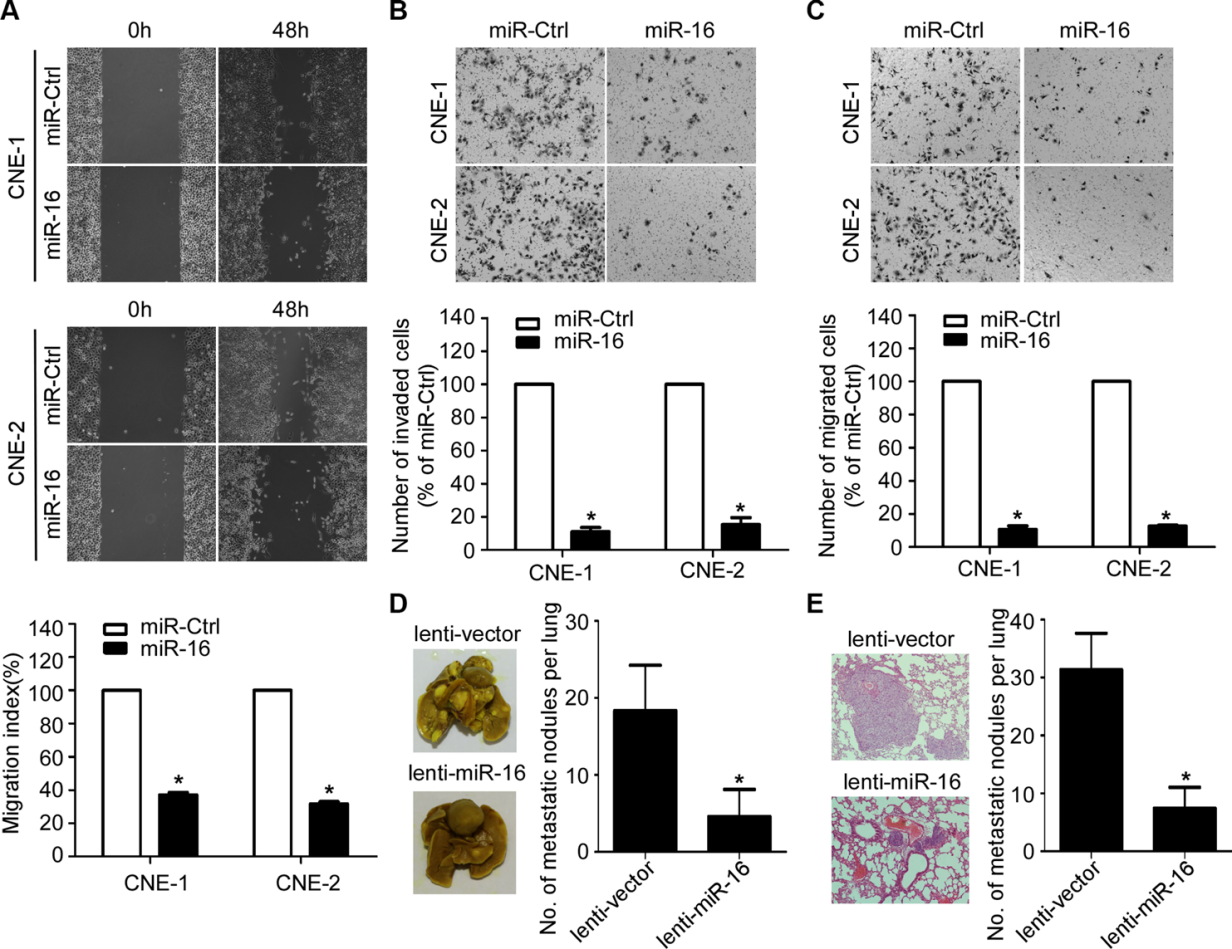
miR-16 inhibits NPC cell migration, invasion, and lung metastatic colonization (**A**) Representative images and quantification of wound healing assays in the indicated cells. (**B–C**) Representative images and quantification of the indicated cells determined by Transwell migration (B) and invasion assays (C). (**D–E**) Lung metastatic colonization models in nude mice were constructed. Representative images and quantification of macroscopic tumor nodules formed on the lung surface (D); as well as of microscopic tumor nodules formed in the lung tissue sections stained with hematoxylin and eosin (E) are shown. The data are presented as the mean ± S.D.; *p* values were calculated using Student's *t*-test.

To further determine whether the ectopic overexpression of miR-16 affects the migratory and invasive abilities of NPC, we performed wound healing, transwell migration, and invasion assays. The wound healing assay showed that both the CNE-1 and CNE-2 cell lines transfected with miR-16 mimics migrated more slowly compared with those transfected with miR-Ctrl (Figure 3A). In addition, ectopic overexpression of miR-16 in NPC cells significantly reduced the number of migratory and invasive cells as determined by transwell migration and invasion assays (Figure 3B and 3C). Furthermore, we constructed a lung metastatic colonization model to examine the effect of miR-16 on lung metastatic colonization formation ability. As shown in Figure 3D and 3E, the macroscopic and microscopic tumor nodules formed in the lungs by lenti-miR-16 cells were significantly fewer and smaller than those formed by lenti-vector cells. These results indicated that miR-16 overexpression can suppress NPC cell migration and invasion as well as lung metastatic colonization and growth.

### FGF2 is a direct transcriptional target of miR-16 in NPC

**Figure 4:**
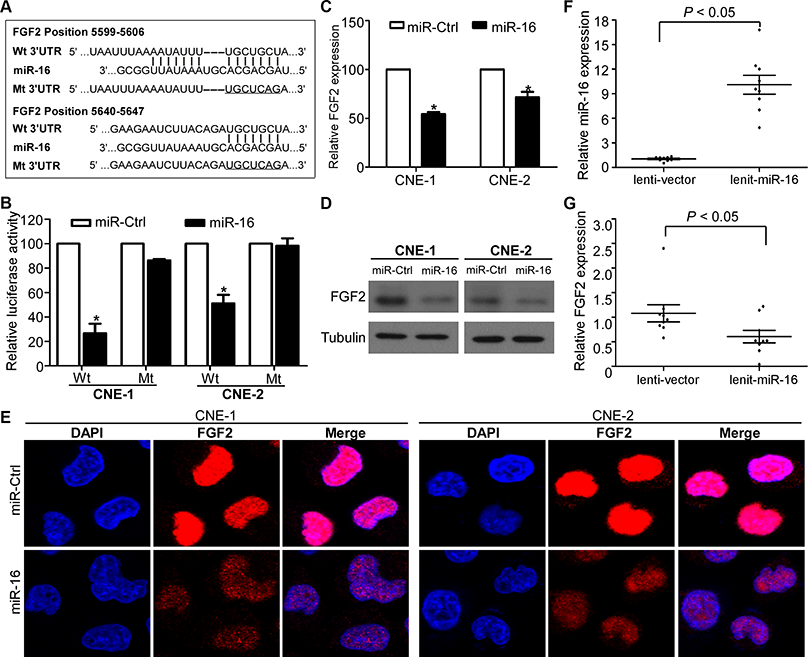
*FGF2* is a direct transcriptional target of miR-16 in NPC (**A**) Wild-type and mutant miR-16 target sequences of the *FGF2* mRNA 3′-UTR. (**B**) Relative luciferase activities of the indicated cells quantified using luciferase reporter assays. (**C–E**) Quantification of *FGF2* mRNA and protein expression using quantitative RT-PCR (C), western blotting (D), and immunofluorescent staining (E). The data are presented as the mean ± S.D.; *p* values were calculated using Student's *t*-test.

Recently, *FGF2* was demonstrated to promote tumor cell proliferation, migration, and invasion [35]. Based on bioinformatics analysis of three online databases (TargetScan, DIANA, and miRanda), the complementary sequence of miR-16 was found in two sites of the 3′-UTR of *FGF2* mRNA (Figure 4A). To validate the transcriptional regulation of miR-16 on *FGF2* expression, we cloned the *FGF2* 3′-UTR regions containing miR-16 binding sites or corresponding mutant sites into a luciferase reporter vector, and we performed luciferase reporter assays in CNE-1 and CNE-2 cells. miR-16 overexpression significantly reduced the luciferase activity of the *FGF2* wild-type reporter plasmids compared with miR-Ctrl, and this inhibition was not observed in *FGF2* mutant reporter plasmids (Figure 4B). Furthermore, miR-16 overexpression significantly decreased *FGF2* expression at both the mRNA and protein levels (Figure 4C–4E). In addition, xenograft tumors formed in mice by lenti-miR-16 cells expressed higher levels of miR-16 and lower levels of *FGF2* mRNA compared with tumors formed in mice by lenti-vector cells (Figure 4F and 4G). These findings suggested that miR-16 can transcriptionally regulate *FGF2* expression in NPC cell lines.

### Silencing of miR-16 promotes NPC cell viability, proliferation, migration, and invasion

**Figure 5:**
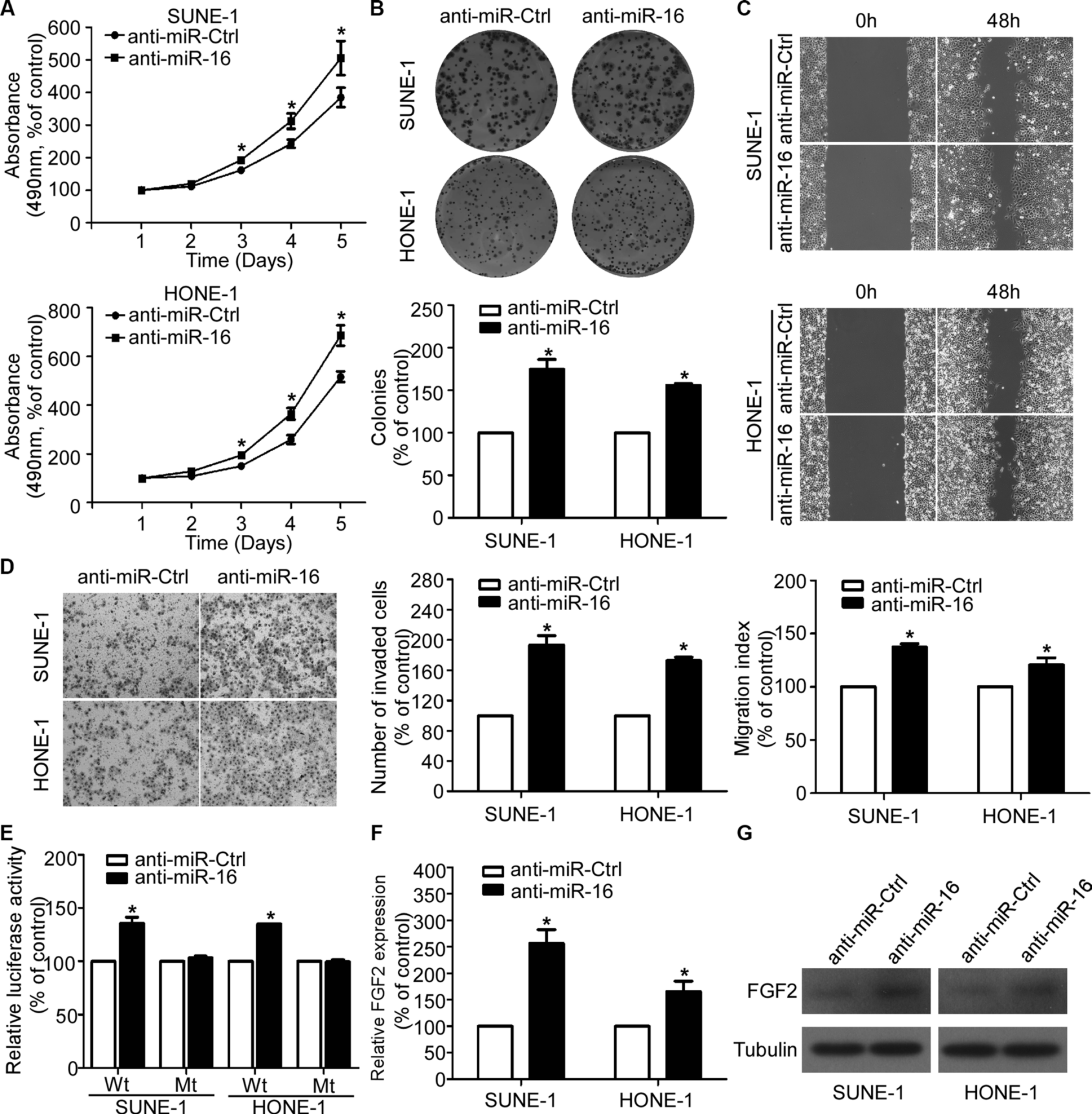
Silencing of miR-16 promotes NPC cell viability, proliferation, migration, and invasion (**A–D**) Effects of the silencing of miR-16 on NPC cell viability, proliferation, migration and invasion. Representative results of the MTT (A), colony formation (B), wound healing (C), and transwell invasion assays (D). (**E**) Relative luciferase activities of the indicated cells quantified using luciferase reporter assays. (**F–G**) Quantification of *FGF2* mRNA and protein expression using quantitative RT-PCR (F), and western blotting (G). The data are presented as the mean ± S.D.; *p* values were calculated using Student's *t*-test.

To explore whether inhibition of miR-16 affects the NPC cell viability, proliferation, migratory, and invasive abilities, we transiently transfected SUNE-1 and HONE-1 cells with miR-16 inhibitors and performed MTT, colony formation, wound healing, and transwell invasion assays. As determined by the MTT and colony formation assays, silencing of miR-16 significantly increased the NPC cell growth and colony formation rates (Figure 5A and 5B). The wound healing assay showed that both SUNE-1 and HONE-1 cells transfected with miR-16 inhibitors migrated more quickly than anti-miR-Ctrl (Figure 5C). The transwell invasion assay also showed that inhibition of miR-16 in NPC cells remarkably increased the number of invasive cells (Figure 5D). Furthermore, inhibition of miR-16 remarkably increased the luciferase activity of the *FGF2* wild-type reporter plasmids, and this increasing was not observed in *FGF2* mutant reporter plasmids (Figure 5E). In addition, silencing of miR-16 obviously increased *FGF2* expression at both the mRNA and protein levels (Figure 5F and 5G).

### miR-16 suppresses the PI3K/AKT and MAPK signaling pathways

**Figure 6:**
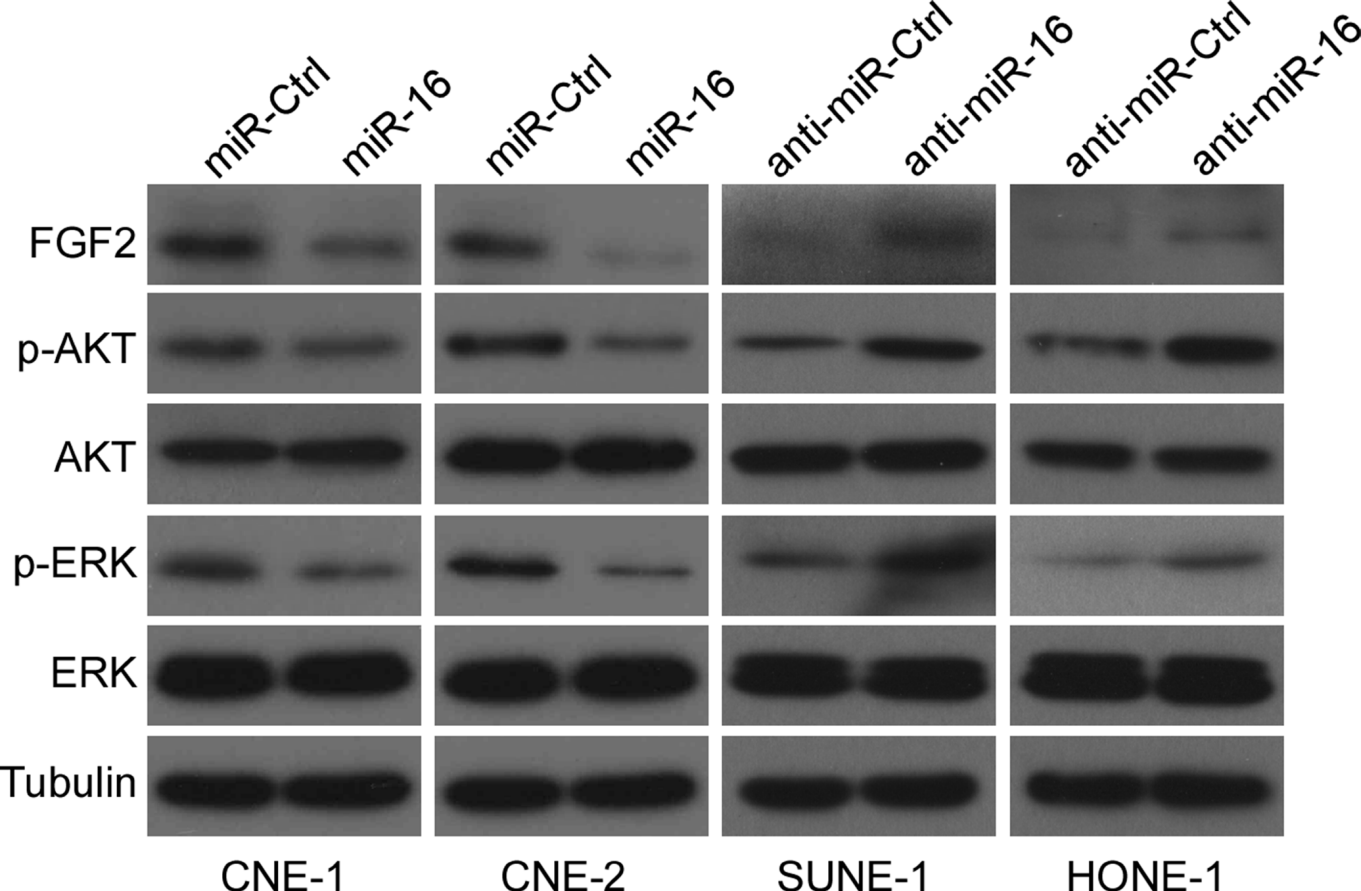
miR-16 suppresses the *PI3K/AKT* and *MAPK* signaling pathways Western blotting was used to determine the expression levels of *p-AKT*, *AKT*, *p-ERK*, and *ERK* in CNE-1 and CNE-2 cells transfected with miR-16 mimics or inhibitor.

*FGF2* is one of the members of the *FGF* family and, it binds to *FGF* receptors (*FGFRs*), thus constituting the *FGF/FGFR* signaling pathway, which can activate several downstream signaling pathways, including the *phosphoinositide-3-kinase/AKT* (*PI3K/AKT*) and the *mitogen-activated protein kinase* (*MAPK*) pathways [35]. Because our findings suggested that miR-16 might inhibit NPC cell proliferation, migration and invasion by targeting *FGF2*, we assessed the effects of miR-16 overexpression on the *MAPK* and *PI3K/AKT* signaling pathways to determine the mechanism by which *FGF2* contributed to NPC progression. Ectopic expression or silencing of miR-16 suppressed or promoted the expression of *p-AKT* and *p-ERK*, but not total *AKT* and *ERK*, in NPC cell lines (Figure 6). These results demonstrated that miR-16 may target *FGF2* to inhibit NPC cell proliferation and invasion via the *MAPK* and *PI3K/AKT* signaling pathways.

### FGF2 mediates the effect of miR-16 on NPC cell proliferation and invasion

**Figure 7:**
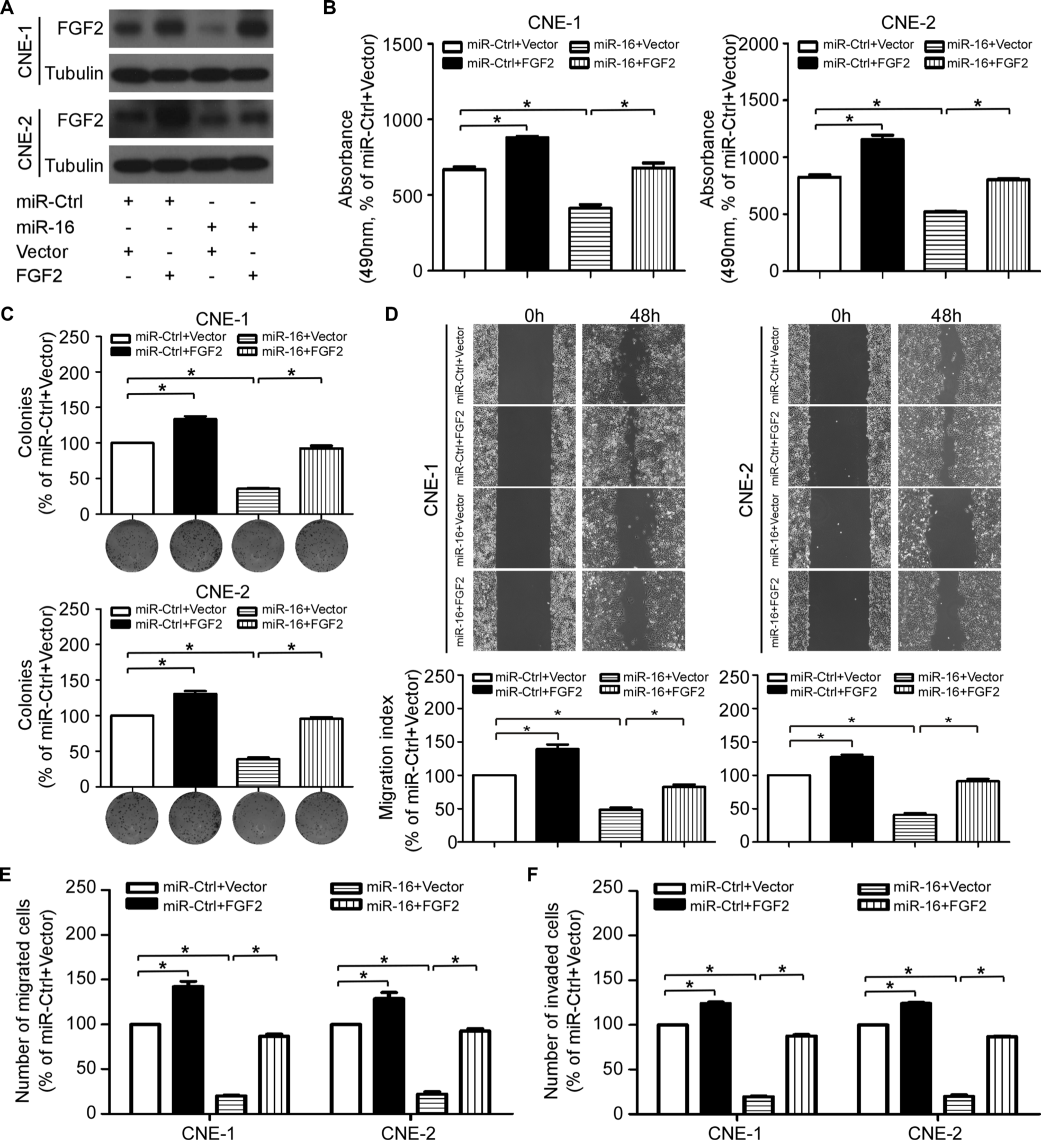
*FGF2* mediates the effect of miR-16 on NPC cell proliferation and invasion (**A**) The *FGF2* protein expression of the indicated cells was determined with western blotting. (**B–F**) Effects of the restoration of *FGF2* on NPC cell viability, proliferation, migration and invasion. Representative results of the MTT (B), colony formation (C), wound healing (D), transwell migration (E) and invasion assays (F). The data are presented as the mean ± S.D.; *p* values were calculated using Student's *t*-test.

To further elucidate whether the tumor suppressive effects of miR-16 in NPC was directly mediated by *FGF2*, we cotransfected CNE-1 and CNE-2 cells with miR-16 mimics or miR-Ctrl together with either the empty vector or the *FGF2* plasmid, which encoded the full-length coding sequence of *FGF2* lacking its 3′-UTR, and we performed MTT, colony formation, anchorage-independent soft-agar, wound healing, and transwell migration/invasion assays. Western blotting validated the expression of FGF2 in the cotransfected cells (Figure 7A). Forced expression of FGF2 abrogated the suppressive effects of miR-16 on cell viability (Figure 7B), proliferation (Figure 7C), migration (Figure 7D and 7E), and invasion (Figure 7F). These results suggested that *FGF2* is a direct and functional target of miR-16 in NPC.

## DISCUSSION

Local recurrence and distant metastasis are the major treatment failure patterns in patients with NPC. Thus, better understanding the mechanisms involved in NPC development and progression are important strategies for improving prognosis. In our current study, we verified that miR-16 is decreased in NPC and that miR-16 targets *FGF2* to inhibit NPC cell proliferation and invasion via the *MAPK* and *PI3K/AKT* signaling pathways.

Previous studies have demonstrated that miRNAs are dysregulated in NPC tissues [7–9], and several dysregulated miRNAs are involved in regulating NPC proliferation, migration and invasion [10–16]. Studies have also suggested that miRNAs can serve as prognostic biomarkers for NPC patients [36]. Our previous microarray data indicated that miR-16 expression was decreased in archived NPC tissue specimens. We confirmed this finding in our present study, and found that miR-16 was downregulated in NPC cell lines except for SUNE-1 and HONE-1, which may due to biological heterogeneity of cancer. We further investigated its biological functions by transient or stable overexpression of miR-16 in NPC cells. Functional investigations showed that forced expression of miR-16 suppressed NPC cell proliferation, migration and invasion *in vitro*, and it inhibited tumor growth and lung metastatic colonization *in vivo*. Interestingly, during the preparation of this manuscript, a study reported that miR-16 could inhibit CNE-2 cell proliferation and increase the apoptosis and radiosensitivity by regulating *Bcl-2* expression [37]. Taken together, these findings indicate that miR-16 plays an important role in NPC development and progression.

miR-16 has been identified as a tumor suppressor in human cancers, and its downregulation may affect tumor cell apoptosis, the cell cycle, cell proliferation, and invasion [38]. miR-16 was firstly reported to be decreased in CLL due to the deletion in chromosome 13q14, and could inhibit apoptosis through targeting *Bcl-2* [17]. miR-16 is decreased in prostate cancer, whereas the expression levels of its targets, namely *Bcl-2*, *CCND1* and *WNT3A*, are inversely increased. Reconstitution of miR-16 expression results in growth arrest, apoptosis, and marked regression of tumor xenograft [18]. In ovarian cancer, *Bmi-1* protein expression has been shown to be regulated by miR-16 and result in ovarian cancer cell proliferation and tumor growth [21]. In non-small cell lung cancer, miR-16 is frequently deleted and downregulated, and miR-16 has been shown to regulate the cell cycle of lung cancer cells by regulating *CCND1* [22]. Here, our *in vitro* and *in vivo* functional study enriched the tumor suppressive role of miR-16 in various types of cancer.

miRNAs exert their function by base pairing to the 3′-UTR of their target genes [1]. Several oncogenes, including *Bcl-2*, *CCND1*, *CCNE1*, *WNT3A*, *Bmi-1*, and *YAP1*, have been identified and confirmed to be targets of miR-16 [17–26]. Each individual miRNA can regulate multiple target genes that harbor target sequence in their 3′-UTR [39]. Therefore, in our present study, we predicted *FGF2* to be a potential target of miR-16 using three online databases, and we then confirmed this prediction using a luciferase reporter assay, quantitative RT-PCR and western blotting. *FGF2*, as a member of the *FGF* family, has also been verified as a target of miR-16 in colorectal carcinoma [40]. *FGF2* has also been demonstrated to be regulated by several other miRNAs, including miR-101, miR-152, miR-497, miR-503, and miR646 [41–45]. *FGF2* binds to *FGF* receptors, thus constituting *FGF*/*FGFR* signaling, which is involved in regulating the tumorigenesis and progression of a variety of cancers [33]. A recent study has also reported that *FGFR1* signaling can be activated by *EBV-encoded latent membrane protein 1* (*LMP1*) and is involved in the EBV-driven pathogenesis of NPC [46]. Our present investigation demonstrated that miR-16 targets *FGF2* to regulate NPC proliferation and invasion via the *MAPK* and *PI3K/AKT* pathways, thereby enriching the understanding of the *FGF/FGFR* signaling pathway involvement in NPC tumorigenesis.

In conclusion, our findings revealed that miR-16, which functions as a tumor suppressor, was decreased in NPC. Ectopic overexpression of miR-16 inhibited NPC cell proliferation, migration, and invasion through targeting *FGF2* to inactivate the *MAPK* and *PI3K/AKT* signaling pathways. These findings demonstrated that the downregulated expression of miR-16 in NPC contributes to the development and progression of NPC. Therefore, miR-16 may serve as a potential therapeutic target for patients with NPC.

## MATERIALS AND METHODS

### Ethics statement

Investigations were performed according to the ethical standards of the Declaration of Helsinki. All research protocols involving patient samples or animals were approved by the Institutional Review Boards of Sun Yat-sen University Cancer Center. Written informed consent was obtained from each patient, and the animal experiments were performed in accordance with the guidelines of the Experimental Animal Care and Use Committee.

### Cell lines and clinical samples

The human immortalized nasopharyngeal epithelial cell line NP69, and six human NPC cell lines (CNE-1, CNE-2, SUNE-1, C666–1, HNE-1, and HONE-1) were available from Sun Yat-sen University Cancer Center (Guangzhou, China). The NP69 cell line was maintained in KSFM (Invitrogen, Grand Island, NY, USA) supplemented with bovine pituitary extract (BD Biosciences, San Diego, CA, USA). The NPC cell lines were cultured in RPMI-1640 medium (Invitrogen) supplemented with 10% FBS (Gibco, Grand Island, NY, USA). All cell lines were incubated in a humidified atmosphere at 37°C with 5% CO_2_. A collection of 16 freshly frozen NPC and 8 normal nasopharyngeal epithelial tissue specimens were obtained from the tissue bank of Sun Yat-sen University Cancer Center (Guangzhou, China).

### RNA isolation and quantitative RT-PCR

Total RNA isolation from NPC cell lines and tissue samples was performed with TRIzol Reagent (Invitrogen) following the manufacturer's instruction. cDNA was synthesized using M-MLV reverse transcriptase (Promega, Madison, WI, USA) and amplified with Platinum SYBR Green qPCR SuperMix-UDG reagents (Invitrogen) using the CFX96 sequence detection system (Bio-Rad, Hercules, CA, USA). The Bulge-Loop miRNA-specific primers (RiboBio, Guangzhou, China) were used for the detection of miR-16. The following primers were used for the detection of *FGF2*: forward, 5′-ACTGGCTTCTAAATGTGTTACG-3′; and reverse, 5′-TTGGATCCAAGTTTATACTGCC-3′). U6 and *GAPDH* were used as controls.

### Transient and stable transfection

The miR-16, inhibitor, or controls (Genepharma, Suzhou, China) were transfected into NPC cells using Lipofectamine 2000 reagent (Invitrogen). The *FGF2* plasmid or empty vector (FulenGen, Guangzhou, China) together with miR-16 mimics or miR-Ctrl was used to cotransfect CNE-1 and CNE-2 cells using Lipofectamine 2000 reagent (Invitrogen). The cells were harvested for assays 48 h after transient transfection. The precursor sequence of miR-16 was synthesized and cloned into the pSin-EF2-puromycin lentiviral plasmid (Addgene, Cambridge, MA, USA) to construct a vector expressing miR-16 (named lenti-miR-16). The empty pSin-EF2 vector (lenti-vector) was used as a control. CNE-1 cells were transfected with lenti-miR-16 or lenti-vector and then selected using puromycin.

### MTT, colony formation, and anchorage-independent soft-agar assays

For the MTT assay, cells (1 × 10^3^) were seeded into 96-well plates and incubated for 0–4 days. On the indicated days, the cells were stained with MTT dye (Sigma, St. Louis, MO, USA), and the absorbance at 490 nm for each well was read on a spectrophotometer. For the colony formation assay, cells (0.5 × 10^3^) were seeded into 6-well plates and cultured for 7 to 12 days. Colonies were fixed with paraformaldehyde, stained with crystal violet, and counted. For the soft-agar assay, cells (2.5 × 10^4^) were suspended in complete medium containing 0.66% agar (Sigma) and then placed on top of a layer of complete medium containing 1% agar in 6-well plates. Colonies were counted under an inverted microscope after 7 to 12 days.

### Wound healing, transwell migration and invasion assays

For the wound healing assay, cells were seeded into 6-well plates and starved for 24 h. Artificial wounds were then created in the cell monolayer with a sterile 200-μl tip, and images were captured at 0 and 48 h with an inverted microscope. For the migration and invasion assays, cells (5 × 10^4^ or 1 × 10^5^) were harvested, resuspended in serum-free medium, and plated into the upper chambers (Corning, Steuben, NY, USA) coated without or with Matrigel (BD Biosciences) on the upper surface of the 8 μm pore size membrane. Medium supplemented with 10% FBS was placed into the lower chambers. After a 16 to 24 h incubation, the cells that had migrated or invaded through the membrane were fixed with paraformaldehyde, stained with crystal violet, and counted under an inverted microscope.

### Tumor models, immunohistochemistry, and hematoxylin and eosin staining

BALB/c-nu mice (4–6 weeks old) were purchased from the Medical Experimental Animal Center of Guangdong Province (Guangzhou, China) and housed in barrier facilities on a 12 h light/dark cycle. For the xenograft tumor growth model, lenti-miR-16 or lenti-vector CNE-1 cells (1 × 10^6^) were subcutaneously inoculated in the right dorsal flank of the mice. Tumor size was measured every 3 days, and tumor volumes were calculated. After two weeks, the mice were sacrificed, and the tumors were excised, weighed, and paraffin-embedded. Sections were cut and subjected to immunohistochemistry with anti-Ki67 (1:400; Proteintech, Wuhan, China) and anti-PCNA (1:400; Proteintech) antibodies. For the lung metastatic colonization model, lenti-miR-16 or lenti-vector CNE-1 cells (1 × 10^6^) were intravenously injected via the tail lateral vein. Five weeks later, the mice were sacrificed, and the lung tissues were excised, paraffin-embedded, cut into slices, and stained with hematoxylin and eosin. The number of macroscopic and microscopic lung tumor nodules was counted.

### Luciferase reporter assay

The FGF2 3′-UTR regions containing predicted miR-16 binding sites and corresponding mutant sites were amplified and then inserted downstream of the luciferase gene in the psiCHECK^™^ vector (Promega). The *FGF2* wild-type or mutant 3′-UTR luciferase reporter plasmids, the p-TK Renilla plasmid (Promega), plus miR-16 mimics, inhibitor, or controls, were used to cotransfect NPC cells using Lipofectamine 2000 (Invitrogen) in accordance with the manufacturer's recommendation. Renilla and firefly luciferase signals were measured 24 h after transfection using the Dual Luciferase Reporter Assay System (Promega).

### Western blotting and immunofluorescent staining

For western blotting, equal amounts of proteins were separated and transferred to PVDF membranes (Millipore, Billerica, MA, USA). The membranes were then incubated with the following primary antibodies: anti-FGF2 (1:1000; Millipore); anti-p-Akt (1:1000; Thr 473; Cell Signaling Technology, Beverly, MA, USA); anti-Akt (1:1000; Cell Signaling Technology); anti-p-ERK (1:1000; Thr 202/Tyr204; Cell Signaling Technology); and anti-ERK (1:1000; Cell Signaling Technology). An anti-α-tubulin antibody (1:1000; Sigma) was used as a protein loading control. For immunofluorescent staining, cells were grown on coverslips (Thermo Fisher Scientific, Rochester, NY, USA) for 24 h and then incubated with an anti-FGF2 antibody (1:200; Millipore) followed by incubation with an Alexa Fluor 594 IgG antibody (Life Technologies, Carlsbad, CA, USA). The cells were then counterstained with DAPI, and images were captured using a confocal laser scanning microscope (Olympus FV1000, Olympus, Tokyo, Japan).

### Statistical analyses

All of the statistical analyses were performed using SPSS 13.0 (SPSS Inc., Chicago, IL, USA). All data shown are representative results of at least 3 independent experiments, and the data are expressed as the mean ± standard deviation (S.D.). Significant differences between two groups were analyzed using two-tailed unpaired Student's *t*-test, and a probability value of *p* < 0.05 was considered significant.
